# Impact of the Ebola outbreak on routine immunization in western area, Sierra Leone - a field survey from an Ebola epidemic area

**DOI:** 10.1186/s12889-017-4242-7

**Published:** 2017-04-26

**Authors:** Xiaojin Sun, T. T. Samba, Jianyi Yao, Wenwu Yin, Lin Xiao, Fuqiang Liu, Xiaoqiang Liu, Jikun Zhou, Zengqiang Kou, Hongwei Fan, Hao Zhang, Aqnes Williams, Paul M. Lansana, Zundong Yin

**Affiliations:** 1 National Immunization Programme, Chinese Center for Disease Control and Prevention, No. 27, Nanwei Road, Beijing, 100050 China; 2 Western Area District Health Management Team, Freetown, Sierra Leone; 3 Emergency Response Center, Chinese Center for Disease Control and Prevention, Beijing, China; 4 Division of Infectious Disease, Chinese Center for Disease Control and Prevention, Beijing, China; 5 Jingzhou Prefecture Center for Disease Control and Prevention, Jingzhou, China; 6 Hunan Provincial Center for Disease Control and Prevention, Changsha, China; 7 Yunnan Provincial Center for Disease Control and Prevention, Kunming, China; 8 Shijiazhuang Prefecture Center for Disease Control and Prevention, Shijiazhuang, China; 9 Shandong Provincial Center for Disease Control and Prevention, Kunming, China; 10 Peking Union Medical College Hospital, Beijing, China; 11 Health News, Beijing, China

**Keywords:** Ebola virus disease, Vaccination coverage, Field survey

## Abstract

**Background:**

Since March 2014, the Ebola Virus Disease (EVD) outbreak in West Africa disrupted health care systems - especially in Guinea, Liberia and Sierra Leone – with a consequential stress on the area’s routine immunization programs. To address perceived decreased vaccination coverage, Sierra Leone conducted a catch-up vaccination campaign during 24–27 April 2015. We conducted a vaccination coverage survey and report coverage estimates surrounding the time of the EVD outbreak and the catch-up campaign.

**Methods:**

We selected 3 villages from each of 3 communities and obtained dates of birth and dates of vaccination with measles vaccine (MV) and the 3rd dose of Pentavalent vaccine (Pentavalent3) of all children under 4 years of age in the 9 selected villages. Vaccination data were obtained from parent-held health cards. We calculated the children’s MV and Pentavalent3 coverage rates at 3 time points, 1 August 2014, 1 April 2015, and 1 May 2015, representing coverage rates before the EVD outbreak, during the EVD outbreak, and after the Maternal and Child Health Week (MCHW) catch-up campaign.

**Results:**

The final sample size was 168 children. MV coverage among age-eligible children was 71.3% (95% confidence interval [CI]: 62.1% - 80.4%) and 45.7% (95% CI: 29.2% - 62.2%) before and during the outbreak of EVD, respectively, and was 56.8% (95% CI: 40.8% - 72.7%) after the campaign. Pentavalent3 coverage among age-eligible children was 79.8% (95% CI: 72.6% - 87.0%) and 40.0% (95% CI: 22.5% - 57.5%) before and during the outbreak of EVD, and was 56.4% (95% CI: 39.1% - 73.4%) after the campaign.

**Conclusions:**

Coverage levels of MV and Pentavalent3 were low before the EVD outbreak and decreased further during the outbreak. Although the MCHW catch-up campaign increased coverage levels, coverage remained below pre-outbreak levels. High-quality supplementary immunization activities should be conducted and routine immunization should be strengthened to address gaps in immunity among children in this EVD-affected area.

## Background

Sierra Leone is in western Africa, is one of the world’s most impoverished countries, and has limited health services resources [1, 2]. Since May 2014, Sierra Leone experienced the largest outbreak of Ebola virus disease (EVD) in history. The outbreak lasted until March 2016, by which time 8,704 EVD cases were confirmed, leading to 3,589 EVD deaths [3, 4]. The Western Rural Area was one of the most severely affected districts in Sierra Leone, EVD cases increased rapidly in this area from August to December 2014, with the confirmation of more than 1,000 cases of EVD [3, 4].

On 8th August 2014, WHO declared the EVD outbreak in West Africa to be a public health emergency of international concern under the International Health Regulations (2005). In response to the outbreak, China’s government offered immediate support, including protective medical clothing, disinfectants, and medicine for the 3 EVD-affected countries. Starting in September 2014, China began to send medical teams with infectious disease experts to the affected African countries to fight the EVD outbreak [5]. The teams consisted of clinicians, laboratorians, and public health trainers, the teams provided medical aid, specimen testing, and training to local health workers about EVD prevention [6].

**Fig. 1:**
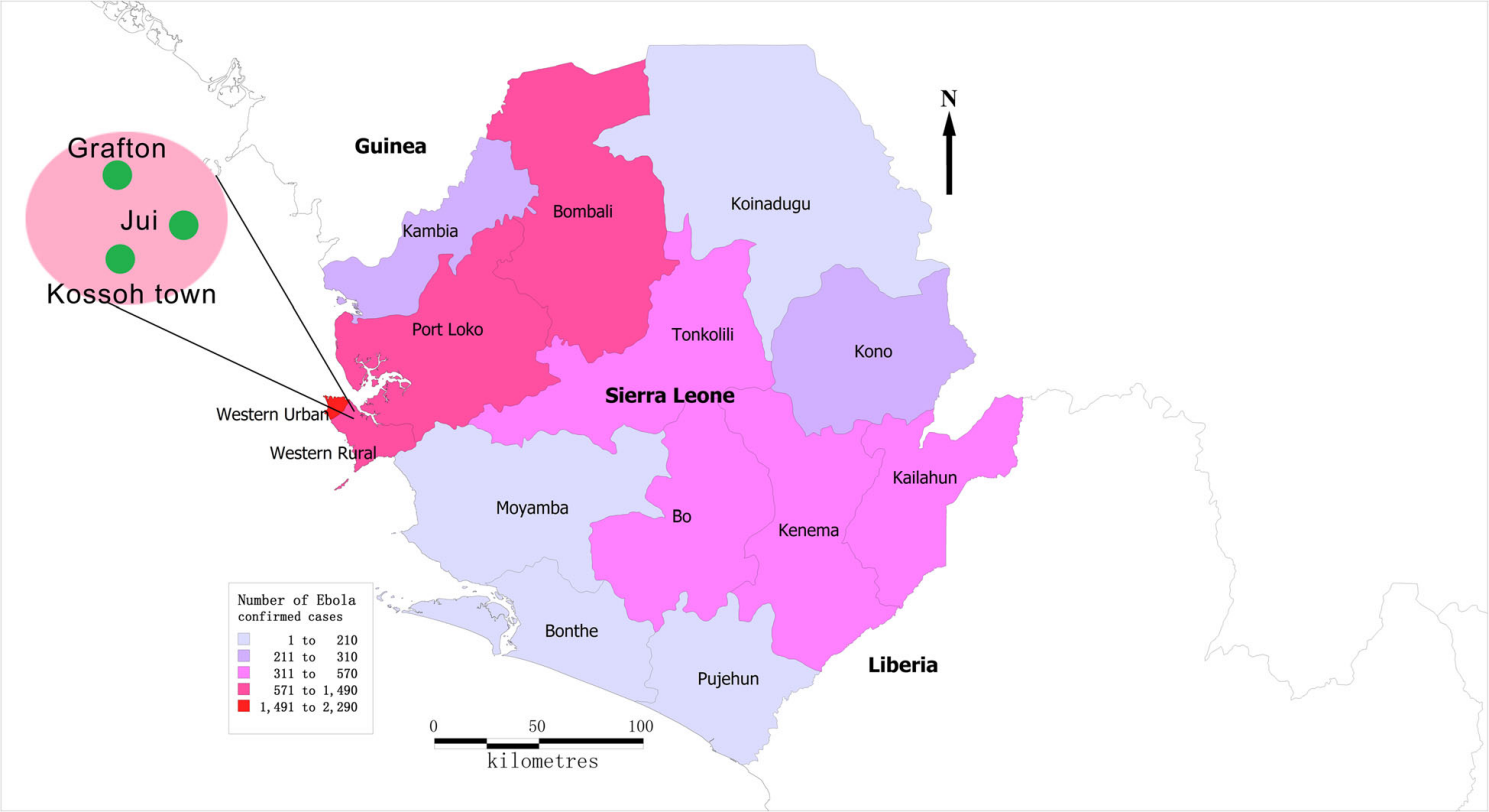
Location of the three villages in Western Area Rural District, Sierra Leone

In Sierra Leone, the China Public Heath Training Team initiated a massive training effort covering 631,680 community residents in 6 districts. The team trained 6016 social mobilizers to prevent EVD spread. In January 2015, the China Public Health Training Team established a pilot program in 3 communities that had the greatest risk of EVD transmission, with a goal to develop a comprehensive model for prevention of EVD in Sierra Leone. The 3 pilot communities were Jui, Kossoh Town, and Grafton, and were in the Western Area Rural District, located in the south-eastern of Freetown, with about 40,000 residents in an area of 10 km^2^. Since January 2015, 14 EVD had been reported in these communities (Fig. 1).

Sierra Leone has insufficient medical resources. Prior to the outbreak, it had a ratio of 1 to 2 doctors per 100,000 population [1]. The Expanded Program of Immunization (EPI) provided 7 different vaccines in 2015, including 1 dose of measles vaccine (MV) at 9 months of age, and 3 doses of diphtheria-tetanus-pertussis/hepatitis B/Haemophilus influenza type b (DTP-HepB-Hib - Pentavalent) at 6, 10, and 14 weeks of age. Studies predicted that due to the EVD outbreak weakening the local health care system, there would be an increase in the number of susceptible children, leading to more deaths from measles and other diseases than from EVD [7, 8]. To reduce the risk of measles and other infectious diseases, Sierra Leone conducted a Maternal and Child Health Week (MCHW) campaign during 24–27 April 2015. The campaign provided vitamin A, albendazole, and catch up vaccination to children who missed any dose of an EPI vaccine, including oral poliomyelitis vaccine and measles vaccine.

To measure EPI vaccine coverage changes associated with the EVD outbreak, and to assess changes of coverage following the MCHW campaign, the China Public Health Training Team conducted a field vaccination coverage survey with the District Health Management Team (DHMT) on 1 May 2015. We report results of this survey of the 3 communities in the Western Area Rural District, Sierra Leone, and provide estimates of coverage before and during the EVD outbreak, and after the catch-up campaign.

## Methods

### Survey population and sampling

The target population of our survey was children under four years of age, which consisted of individuals with birth dates between 1 May 2011 and 30 April 2015. Sampled children resided in 1 of the 3 communities served by the China Public Health Training Team, and whose parents had a Child Health Card (containing immunization, vitamin A and deworming information) for the child. Children whose parents did not have cards were excluded. We selected at random 3 villages from each of the 3 communities, for a total of 9 villages from all 35 villages in the communities (there were 13 villages in Jui, 5 in Kossoh Town, and 17 in Grafton). We included all eligible children from these villages (Fig. 1) [9].

### Data collection

Following training and with assistance and guidance from local volunteers, members of the China Public Health Training Team went house-to-house and obtained information from the parents of children in the target age group (Fig. 1) [9]. We obtained oral consent to collect dates of birth, dates of receiving measles vaccine (MV), and dates of receiving the 3rd dose of Pentavalent vaccine from the Child Health Card.

### Definition of vaccination status

**Fig. 2:**
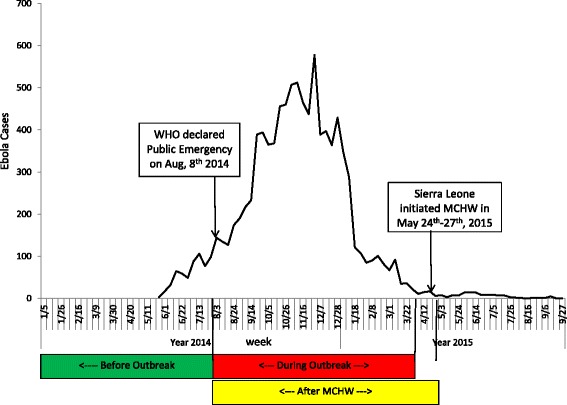
The three time points for vaccination coverage analysis and the epidemiology curve of EVD in Sierra Leone

A child over 9 months of age with documented receipt of MV was considered measles-vaccinated, and a child over 14 weeks of age with documented receipt of the third dose of Pentavalent vaccine was considered Pentavalent3-vaccinated. We determined children’s MV and Pentavalent3 coverage rates at 3 points in time: 1 August 2014, 1 April 2015, and 1 May 2015, representing vaccine coverage levels before the EVD outbreak, during the EVD outbreak, and after the MCHW campaign (Fig. 2) [10].

### Data analysis

Data were recorded with Microsoft Excel (Version 2007). Vaccine coverage rates and rate differences and their 95% confidence intervals were calculated with SAS software (Version 9.4) using Pearson Chi-square test for statistical significance testing.

## Results

### Sample size and demographics

The final sample size was 168 children, parents of all age-eligible children were interviewed. The proportion of children without a Child Health Card was less than 10% (e.g., 5/55 in Grafton). There were 62, 50, and 56 children from the communities of Jui, Grafton, and Kossoh Town, respectively. There were 48, 47, 40 and 33 children in the age groups of 0–1, 1–2, 2–3 and 3–4 years old, respectively. There were 94 boys and 74 girls in the sample. For determining MV-vaccinated and Pentavalent3-vaccinated, the numbers of age-eligible children were 94 and 119, respectively (Table 1).

**Table 1:** Number of children over 9 months and 14 weeks old in the 3 study phases (before the EVD outbreak, during the EVD outbreak, and after the catch-up campaign)

Ages	Items	Phase 1 (Before Aug1, 2014)	Phase 2 (Aug 2, 2014 – Apr 1, 2015)	Phase 3 (Aug 2, 2014 – May 1, 2015)
≥9 M	DoB^a^ covered	May 1, 2011- Oct 31, 2013	Nov 1, 2013- Jul 1, 2014	Nov 1, 2013- Aug 1, 2014
	No. of Children	94	35	37
≥14 W	DoB^a^ covered	May 1, 2011- Apr 25, 2014	Apr 26, 2014- Dec 22, 2014	Apr 26, 2014- Jan 22, 2015
	No. of Children	119	30	32

^a^Note: DoB = Date of birth

### Coverage rates and changes by time

In phase 1 (before EVD), the age-eligible, MV-vaccinated rate was 71.3% (95% CI: 62.1% - 80.4%) while in phase 2 (during EVD), the MV-vaccinated rate was 45.7% (95% CI: 29.2% - 62.2%). Therefore, age-eligible coverage during the EVD outbreak was 25.6 percentage points (95% CI: -44.2 to −7.0 percentage points) lower than before the EVD outbreak (*χ* ^*2*^ = 7.3, *P* < 0.01). Following the MCHW campaign, MV coverage increased to 56.8% (95% CI: 40.8% - 72.7%), but was not statistically different than MV coverage during the EVD outbreak (*χ* ^*2*^ = 0.9, *P* > 0.05) (Table 2).

**Table 2:** Age-eligible MV and Pentavalent3 coverage and changes by phases (before the EVD outbreak, during the EVD outbreak, and following the catch-up campaign)

	Phase 1	Phase 2	Changes (Phase 2–1) (95% CI)	Phase 3	Changes (Phase 3–2) (95% CI)
	No. of vaccinated/ No. of investigated (Coverage 95% CI)	No. of vaccinated/ No. of investigated (Coverage 95% CI)		No. of vaccinated/ No. of investigated (Coverage 95% CI)	
MV					
Jui	32/37 (86.5, 75.5–97.5)	8/13 (61.5, 35.1–88.0)	−25.0 (−50.2, 0.3)	8/13 (61.5, 35.1–88.0)	0.0 (−37.4, 37.4)
Grafton	15/28 (53.6, 35.1–72.0)	1/9 (11.1, 0–31.6)	−42.5 (−79.7, −5.3)	4/10 (40.0, 9.6–70.4)	28.9 (−10.8, 68.5)
Kossoh town	20/29 (69.0, 52.1–85.8)	7/13 (53.9, 26.8–81.0)	−15.1 (−46.5, −16.2)	9/14 (64.3, 39.2–89.4)	10.4 (−26.7, 47.5)
Subtotal	67/94 (71.3, 62.1–80.4)	16/35 (45.7, 29.2–62.2)	−25.6 (−44.2, −7.0)	21/37 (56.8, 40.8–72.7)	11.1 (−12.1, 34.1)
Pentavalent3					
Jui	39/45 (86.7, 76.7–96.6)	6/9 (66.7, 35.9–97.5)	−20.0 (−46.7, 6.7)	7/10 (70.0, 41.6–98.4)	3.3 (−38.5, 45.2)
Grafton	22/35 (62.9, 46.9–78.9)	1/10 (10.0, 0–28.6)	−52.9 (−88.0, −17.7)	3/10 (30.0, 1.6–58.4)	20.0 (−15.1, 55.1)
Kossoh town	34/39 (87.2, 76.7–97.7)	5/11 (45.5, 16.0–74.9)	−41.7 (−69.4, −14.0)	8/12 (66.7, 40.0–93.3)	21.2 (−19.3, 61.8)
Subtotal	95/119 (79.8, 72.6–87.0)	12/30 (40.0, 22.5–57.5)	−39.8 (−57.8, −21.8)	18/32 (56.3, 39.1–73.4)	16.3 (−8.6, 41.1)

In phase 1 (before EVD), the age-eligible Pentavalent3-vaccinated coverage was 79.8% (95% CI: 72.6% - 87.0%), while in phase 2 (during EVD), Pentavalent3-vaccinated coverage was 40.0% (95% CI: 22.5% - 57.5%). Therefore, age-eligible coverage during the EVD outbreak was 39.8 percentage points (95% CI: -57.8 to −21.8 percentage points) lower than before the EVD outbreak (*χ* ^*2*^ = 18.8, *P* < 0.01). Following the MCHW campaign, Pentavalent3 coverage increased to 56.3% (95% CI: 39.1% - 73.4%), but was not statistically different than coverage during the EVD outbreak (*χ* ^*2*^ = 1.6, *P* > 0.05) (Table 2).

## Discussion

Our survey was conducted in the Western Area Rural District of Sierra Leone where EVD was epidemic [9]. It was the first field survey conducted by the China Public Health Training Team in Sierra Leone, and was conducted during the later period of the EVD outbreak, with the intent to evaluate the impact of the EVD outbreak on childhood immunization. Based on 2 key events - the declaration of a public health emergency of international concern and a catch-up vaccination and health campaign - we divided the study into 3 phases for analysis: before the EVD outbreak (phase 1), during the outbreak (phase 2), and after the MCHW catch-up campaign (phase 3). We conducted a parent-interview, record-verified survey to determine measles and Pentavalent vaccination coverage rates for these 3 points in time.

We found that the MV and Pentavalent3 vaccination coverage rates were similar to each other, with the rates of 71.3% and 79.8% respectively, reflecting that coverage was relatively low before EVD outbreak. There had been supplementary immunization activities (SIAs) conducted in Sierra Leone every 3 years since 2003 [11, 12], but no children in our survey were included in the most recent SIA, which was conducted in May 2012, before the EVD outbreak. A previous survey showed that MV coverage was 69.0% in Sierra Leone, which was lower than coverage that had been reported to WHO [11, 12]. There was a gap between the current routine coverage and the targets in the WHO Regional strategic plan for DTP3 coverage to be at least 90% and MV coverage to be at least 95% by 2020 [13, 14]. The reasons for low coverage were thought to be a weak economy and insufficient of health care staff [15, 16].

We showed that between the time prior to the WHO declaration that the EVD outbreak was a public health emergency of international concern and the EVD outbreak, MV coverage decreased by 25.6 percentage points (from 71.3% to 45.7%) in three communities. This figure was consistent with modeling impact scenarios by Takahashi and colleagues [7]. Under the scenario of EVD lasting for one year with MV coverage lowered by 25%, there would be more than 800,000 children under five years old who missed their MV vaccination in Western African countries [7] – clearly a cause for great concern. Reasons for the large decrease that we measured (from 71.3 to 45.7) may include interruption of routine childhood immunization services, fear among local residents of being infected and refusing to go to a health unit for vaccination, or local health workers being too busy fighting EVD [15, 17]. The decreases in coverage that we measured ranged from 15.1% in Kossoh Town to 42.5% in Grafton, indicating that the decrease was variable, and that some areas could be at greater risk of measles outbreaks than other areas.

Similarly, we showed that coverage of Pentavalent3 decreased by 39.8 percentage points (from 79.8% to 40.0%), greater than the MV decrease. Again, there was a gap between measured coverage and the Regional strategic plan goal that DTP3 should be at least 90%. We believe that the decrease may be due to the same reasons for the MV coverage decrease. According to the global and regional immunization profile, there were 1654 diphtheria, 1287 tetanus and 9354 pertussis cases in the African Region in 2015, revealing a risk of epidemics and outbreaks [18]. A high proportion of susceptible children place the childhood population under the threat of diphtheria, tetanus, and pertussis.

The China Public Health Training Team worked to support the DHMT, Western Area for the EVD control and prevention. The China Medical Team for clinical treatment was also based in Western Area, Sierra Leone. We felt that this support would delay disruption of the local health system by EVD compared with other areas. The MV coverage rate after the MCHW campaign increased by 11.1%, while Pentavalent3 coverage increased by 16.3%, but even with these increases, coverage was lower than before the EVD outbreak, showing a persistent gap between current coverage and the Regional plan targets [14]. Thus, the goals of the MCHW campaign increase coverage quickly and significantly, especially for children who missed one or more doses of vaccine, were not fully achieved. We speculate that the main reason for the lower coverage after the MCHW campaign may be because healthcare workers were unable to reach every child in a short period of time.

Our survey has several limitations. First, we only collected information from children whose parents had Child Health Cards. For those without cards, the possibility of missing vaccination maybe higher, so we might have overestimate vaccination coverage. Because the cards were essential for obtaining medical services, parents tend to keep cards carefully and the proportion of lost cards is likely to be low. Second, there were no EVD cases since Mach 2015 in the three communities, but EVD cases were reported continuously in Sierra Leone, especially in western urban areas. Therefore, the results from our survey following the MCHW campaign might not represent coverage at the end of the Sierra Leone EVD outbreak. Third, our survey was conducted in only 3 communities in Western Rural Area of Sierra Leone and the sample was small. Therefore, the survey might represent only similar rural areas and generalized only with caution.

We conclude that routine vaccination coverage was low, even before the epidemic of EVD. The outbreak of EVD put the local health system under significant strain and further decreased coverage. The MCHW campaign did increase coverage but not to the level seen before EVD outbreak. In order to prevent vaccine preventable diseases, such as measles, we suggest that high quality SIAs of MV should be conducted in the EVD epidemic areas for children under 5 years of age to rapidly achieve and maintain high coverage and immunity [16]. Field supervision should be conducted to identify lower coverage areas for further improvement during SIAs [13], and one more dose of MV should be added to routine immunization. In addition, due to the gap between coverage in Sierra Leone and the target of the strategic plan of WHO, routine immunization should be strengthened as a cornerstone for sustainable measles control [16]. Accurate estimates of the target population, training for health workers, cold chain equipment, injection safety, and adverse events management should be considered [19]. As WHO commended Sierra Leone for stopping EVD virus transmission in November 2015, we recommend that more financial resources, including domestic and international support, should be put into the health system, and that human resources and healthcare workers should be expanded and improved, especially in the district and peripheral health unit levels.

## Conclusions

Coverage of MV and Pentavalent3 were relatively low before the EVD outbreak and decreased further during the outbreak. Although the MCHW catch-up campaign increased coverage levels, coverage remained lower than that in the pre-outbreak. High-quality supplementary immunization activities should be conducted and routine immunization should be revised and strengthened.
